# Cohesion, order and information flow in the collective motion of mixed-species shoals

**DOI:** 10.1098/rsos.181132

**Published:** 2018-12-12

**Authors:** Ashley J. W. Ward, T. M. Schaerf, A. L. J. Burns, J. T. Lizier, E. Crosato, M. Prokopenko, M. M. Webster

**Affiliations:** 1 School of Life and Environmental Sciences, University of Sydney, Sydney, Australia; 2 School of Science and Technology, University of New England, Armidale, Australia; 3 Taronga Conservation Society Australia, Sydney, New South Wales, Australia; 4 Complex Systems Research Group and Centre for Complex Systems, Faculty of Engineering and IT, University of Sydney, Sydney, Australia; 5 School of Biology, Harold Mitchell Building, St Andrews, Fife KY16 9TF, UK

**Keywords:** aggregation, shoaling, schooling, collective behaviour, transfer entropy, mixed-species groups

## Abstract

Despite the frequency with which mixed-species groups are observed in nature, studies of collective behaviour typically focus on single-species groups. Here, we quantify and compare the patterns of interactions between three fish species, threespine sticklebacks (*Gasterosteus aculeatus*), ninespine sticklebacks (*Pungitius pungitius*) and roach (*Rutilus rutilus*) in both single- and mixed-species shoals in the laboratory. Pilot data confirmed that the three species form both single- and mixed-species shoals in the wild. In our laboratory study, we found that single-species groups were more polarized than mixed-species groups, while single-species groups of threespine sticklebacks and roach were more cohesive than mixed shoals of these species. Furthermore, while there was no difference between the inter-individual distances between threespine and ninespine sticklebacks within mixed-species groups, there was some evidence of segregation by species in mixed groups of threespine sticklebacks and roach. There were differences between treatments in mean pairwise transfer entropy, and in particular we identify species-differences in information use within the mixed-species groups, and, similarly, differences in responses to conspecifics and heterospecifics in mixed-species groups. We speculate that differences in the patterns of interactions between species in mixed-species groups may determine patterns of fission and fusion in such groups.

## 1. Introduction

Group-living is a widespread strategy among animals. For many grouping species, grouping can increase the efficiency of foraging, or anti-predator behaviour. Less widespread benefits include access to mates, resource conservation and improved navigation (see [1,2]) for reviews of the benefits of grouping). Although most discussions of grouping behaviour in animals focus on single-species assemblages, mixed-species groups are a common phenomenon in nature [3]. Examples of mixed-species groups have been recorded in songbirds [3–11], in artiodactyls, primates (including mixed-species groups of ungulates and primates) and in other mammalian orders (reviewed by Stensland *et al*. [12]) and in fishes [13–16]. In order to differentiate between simple, resource-based assemblages of multiple species and true mixed-species groups, we follow Goodale *et al.* [17] and define mixed-species groups as occurring where members of different species simultaneously associate and interact with both conspecifics and heterospecifics. Associating with heterospecifics in a mixed-species group can provide individuals with significant benefits. These potentially include basic risk-dilution benefits of associating with others, improved predator detection capabilities in mixed-species groups [18,19], and increasing foraging opportunities based either on the spread of information on foraging opportunities among species [20,21], or facilitation effects, such as the flushing of the prey of one species by the activity of another [6]. In some cases, associating with heterospecifics can reduce the competition costs associated with foraging in groups due to niche divergence between species; however, the competition costs between species are often asymmetric [22,23]. Broadly speaking, mixed-species groups do tend to be formed by species that exploit similar resources and which have overlap in locomotion and/or activity patterns [24–26]. Mixed-species groups are often skewed in terms of the relative numbers of different species that comprise them. Studying mixed-species fish shoals in Canadian lakes, Hoare *et al*. [14,15] reported groups consisting of one or two numerically dominant core species accompanied by a small number of representatives of minority species.

The coherent and synchronous movements of animal groups, often referred to as collective motion, emerges from the repeated interactions of group members [27–29]. To date, these interactions have been considered almost exclusively in the context of single-species aggregations. To develop a deeper understanding of mixed-species groups, it is important that we examine how conspecifics and heterospecifics interact within mixed-species groups and indeed whether single-species groups are characteristically different from mixed-species groups, particularly in terms of two key descriptors of collective motion, cohesion and order. Group cohesion describes the distance between individuals within the group, while order can refer to the alignment of individuals across the group when it is measured as polarization. Those groups that express high group cohesion and polarization may move more efficiently and maximize the benefits of grouping for individual group members [30].

In addition to these measures, a particular feature of collective behaviour is the propagation of information across a group that occurs when a single individual initiates a change in direction and, with some small time delay, its near neighbours respond by adapting their trajectories to follow suit. Gradually, the information about the change in direction propagates throughout the group until all group members are following the new trajectory. Information theoretic measures are increasingly being used to provide a more rigorous framework for the examination of information transfer in biological systems. In particular, transfer entropy quantifies the reduction in uncertainty in predicting the updates of one time series (in this case, the temporal sequence of spatial movements of an animal) that can be achieved by knowing the past values of a second time series (a similar sequence derived from a second individual animal). Effectively, it models the directed effect of one time series (the source) on another (the target), from which we can infer the information flow from source to target [31,32]. Transfer entropy has previously been applied to analyse information flow in models [33] and data collected from collective interactions, including in bats [34], soldier crabs [35], fish [36,37], insects [38], robotic soccer players [39] and indeed in interactions between fish and robots [40]. Such analyses have revealed, for example, strong wave-like sequences of information propagating across a group as the group undertakes collective turns [33,37].

We examined first, the occurrence of mixed-species shoals of fish in the wild among three common shoaling species, the threespine sticklebacks (*Gasterosteus aculeatus*), ninespine sticklebacks (*Pungitius pungitius*) and roach (*Rutilus rutilus*). We then subsequently examined collective motion within single-species and mixed-species shoals under controlled conditions, in an arena. Since single-species groups are most likely to comprise individuals with similar motivations and patterns of activity (e.g. [26]), we predicted that, compared to mixed-species shoals, single-species shoals would be more cohesive and polarized with higher levels of information transfer among group members. Furthermore, threespine sticklebacks are more similar to ninespine sticklebacks in appearance and ecology than they are to roach, which might act to promote association between the stickleback species to a greater extent than between the sticklebacks and the roach. Based on this, we further predicted that mixed-species shoals comprising two stickleback species would be more cohesive and polarized with greater information transfer than mixed-species shoals comprising stickleback and roach.

## 2. Methods

### 2.1. Field survey

We performed a pilot field survey in July 2016 to determine whether the fish at our study site (a stretch of the Great Eau, a river in the East Lindsey district of Lincolnshire, UK, (53°22'10.83″ N, 0°11'21.96″ E)) form into mixed-species shoals. During summer and autumn periods at this site, there are large numbers of juveniles of three shoaling species of fish: threespine sticklebacks [41], ninespine sticklebacks and roach. To perform our survey, we used four GoPro Hero3+ cameras, each fixed to aluminium tubing, which allowed the camera to sit on the river bed, at a depth of approximately 1 m. The cameras were spaced along the river, approximately 8 m apart. In total, we filmed for 6 h per camera, spread over 2 days (24 h of video footage in total). From the resulting video, we analysed 1 min of film from each camera every 20 min. We synchronized the cameras so that we used the same minute in time, thus reducing the risk of counting the same shoals more than once. We allowed 20 min between our 1 min sampling periods to allow for the fish to move on, or for shoals to break up and reform. Although we readily acknowledge that this method cannot exclude the possibility of pseudoreplication, it was sufficient for the purpose of determining the presence or the absence of naturally occurring single- and mixed-species shoals. From the film, we noted each shoal that passed the camera (where a shoal is defined as an aggregation of two or more fish, moving in the same or similar direction and where each individual is within approximately four body lengths of its nearest neighbour (following [42]), and recorded the number and species of fish in each shoal.

### 2.2. Experimental studies in an arena

In October 2016, we returned to the Great Eau to conduct experiments on single- and mixed-species shoals of the three species of fish described.

### 2.3. Study animals

We collected our three species of fish using large handnets from the Great Eau at 53°19′016 N, 0°08′16 E. We measured water temperatures at the capture sites with a digital thermometer (Traceable Digital Thermometer, Control Company, Friendswood, TX, USA). The fish were transferred to nearby holding facilities, where each species was maintained separately in round plastic containers (0.9 m diameter, 0.25 m water depth). Fish were held for 16–20 h before they were used in experiments. The temperature of the water in the vats was ambient and ranged from 14.8°C to 15.4°C, which is similar to our measurements of the river at the time of capture (14.6°C to 14.9°C). The fish were not fed while in captivity. They were released at their site of capture following completion of the experiments, approximately 24 h following their initial capture.

### 2.4. Experimental protocol

All experiments were conducted in a circular, white plastic arena (0.9 m in diameter), filled to a depth of 16 cm. We placed a circular white plastic bowl (0.32 m in diameter) in the centre of the arena to create an annulus. The arena was lit using two 10 W LED strips positioned at either side of the arena. We used white plastic sheeting to screen the arena and thereby minimize external disturbance to the experimental animals. We used a Panasonic Lumix GH4 positioned 1.2 m above the arena to film the experiments at a resolution of 1080p and 50 fps.

At the beginning of each trial, we captured six fish from our holding tanks and transferred them in a 0.5 l vessel to the experimental arena. The fish were size matched by eye prior to each trial. The fish were then allowed to move freely throughout the arena for the following 8 min. The fish were then removed and replaced with six new fish. Each fish was used once. We conducted five separate treatments consisting of three single-species shoaling treatments (threespine sticklebacks ‘3SS’; ninespine sticklebacks ‘9SS’; roach ‘R’) and two mixed-species shoaling treatments, using three individuals of each of two species (threespine sticklebacks with ninespine sticklebacks ‘3SS–9SS’, and threespine sticklebacks with roach ‘3SS–R’). We carried out six replicates of each treatment, a total of 30 replicates. At the end of each trial, all of the fish were measured from still photographs taken following each replicate. Across all groups, body length was 30.4 ± 2.96 mm (mean ± s.d.), and there were no significant differences between treatments in relation to the body lengths of the fish used (ANOVA: *F*_4,175_ = 0.66, *p* = 0.62).

### 2.5. Data extraction and preparation

We used 5 min of video footage for analysis, resulting in a series of 15 000 time steps for each replicate. To standardize across replicates and across treatments, we used the time period from 2:30 min to 7:30 min in each video. This was converted to AVI format using VirtualDub and then tracked using CTrax tracking software [43]. From the resulting trajectories, we calculated the mean of the median speeds of each fish (median speeds are used due to the right skew typically seen in speed distributions), the mean distance between all fish and the mean polarization of the group during each trial, using methods described in detail in [44].

Transfer entropy was calculated on heading updates and differences for each pair of individuals within each group, across all relevant samples. Transfer entropy is based on conditional mutual information (CMI) [45]: the CMI from *X* to *Y* given *Z* tells us how much information a sample *x* of the variable *X* tells us about the co-sample *y* of *Y*, given that we know the value of co-sample *z* of the variable *Z*. In other words, this is a log-ratio of probability of *y* given *x* and *z* versus given *z* alone:

$$I(X;Y|Z)=\;\left\langle \log\frac{\;p(y|x,z)}{\;p(y|z)}\right\rangle .$$

As detailed in [37], we calculated transfer entropy between fish as a CMI about the target's current heading update gained from the relative heading of the source, given (a vector of the *k*) previous relative headings of the target. Samples for the calculation are taken from all relevant directed pairs of the appropriate type (e.g. for the overall analysis we used all possible pairs, then additionally for the mixed-species groups we used pairs within a single species, and also pairs comprising the different species) at every time step in the trial. Specifically, we used the KSG estimator [46] from the JIDT open-source software [47] with four nearest samples used in the search space, and an embedding history length for target of *k* = 3 (with embedding delay *τ* = 1) selected to maximally remove (bias-corrected) stored information in the target being misattributed as transfer [48] as averaged across all fish, and a source-target time delay of *u* = 5 (10 ms) selected to optimize the transfer detected [49]. This then allowed us to determine mean pairwise transfer entropy for each group, or in the case of mixed-species groups, to determine mean transfer entropy within and between species. For each individual trial, we calculated the surrogate distribution of the mean transfer entropy under the null hypothesis that there was no (directed) relationship between source and target (see [47] describing techniques in [50,51]). For an average transfer entropy estimated from *N* samples, each surrogate transfer entropy is estimated by resampling the source value for each of the *N* samples, then computing the new average transfer entropy over the new surrogate samples. The transfer entropy estimate can then be compared to the surrogate distribution to test whether there is a statistically significant directed relationship.

In addition, we examined the mean distance of threespine sticklebacks to conspecifics and to ninespine sticklebacks in the 3SS–9SS treatment, and to conspecifics and to roach in the 3SS–R treatment. We also calculated the mean position of fish in the shoal according to their travelling order. Fish leading the shoal were given a score of 1, the next was given a score of 2 and so on to the last fish, which was given a score of 6. We then compared the mean position of each species in the 3SS–9SS treatment and again in the 3SS–R treatment.

Subsequently, we analysed the interactions between animals within the single- and mixed-species groups, as described in detail in a previous paper [44]. In particular, we examined the positions and orientation of near neighbours relative to a focal fish at each time step. In both cases, the coordinates were transformed such that the focal fish was positioned at the origin (*0*,*0*) and travelling parallel to the *x*-axis in a positive direction. This process was repeated taking each fish within the group as the focal fish in turn and we then constructed heat plots based on the data gathered. In addition, we used these data to produce graphs to show how the fish, on average, adjusted their heading (or angle of motion) as a function of the relative *y*-coordinate of partner fish, which is a key aspect of the rules of motion, or interaction, that are proposed to govern how animals respond to near neighbours during collective motion [28,29].

### 2.6. Statistical analysis

We analysed the results in R [52], examining for departures from normality using Shapiro–Wilks tests and the equality of variances using Levene's test. Where Levene's test indicated inequality of variances, we log transformed the data prior to analysis, although untransformed data are presented in the figures. We used the lme package in R to analyse the groups and used planned, non-orthogonal contrasts [53] to compare (i) single-species groups, (ii) mixed-species groups, (iii) 3SS, 9SS and 3SS–9SS and (iv) 3SS, R and 3SS–R. Subsequently, we compared the behaviour of the different species within the mixed shoals, using paired *t*-tests in the case of measures of position and neighbour distance and a linear mixed-effects model in the case of measures of transfer entropy. In the latter case, we used the source species and the target (receiver) species as fixed effects, and included the trial group as a random effect to account for the non-independence of individuals within groups.

## 3. Results

### 3.1. Field survey

We observed a total of 57 shoals, including single-species shoals of 3SS, 9SS and R, and mixed-species 3SS–9SS and 3SS–R shoals (table 1).

**Table 1.** Results of field survey, showing the mean (±s.d.) number of each species of fish in 57 shoals captured on video. 3SS = threespine sticklebacks, 9SS = ninespine sticklebacks.

species composition	number of shoals observed	mean (±s.d.) number of 3SS	mean (±s.d.) number of 9SS	mean (±s.d.) number of roach
single species, 3SS	31	9.2 (7)		
single species, 9SS	3		4.3 (2.3)	
single species, roach	11			16.7 (8.9)
mixed 3SS and 9SS	4	12.3 (7.4)	2.5 (0.6)	
mixed 3SS and roach	8	11.6 (8.2)		2.5 (0.9)
mixed 9SS and roach	0			
mixed 3SS, 9SS and roach	0			

### 3.2. Arena experiments

**Figure 1.**
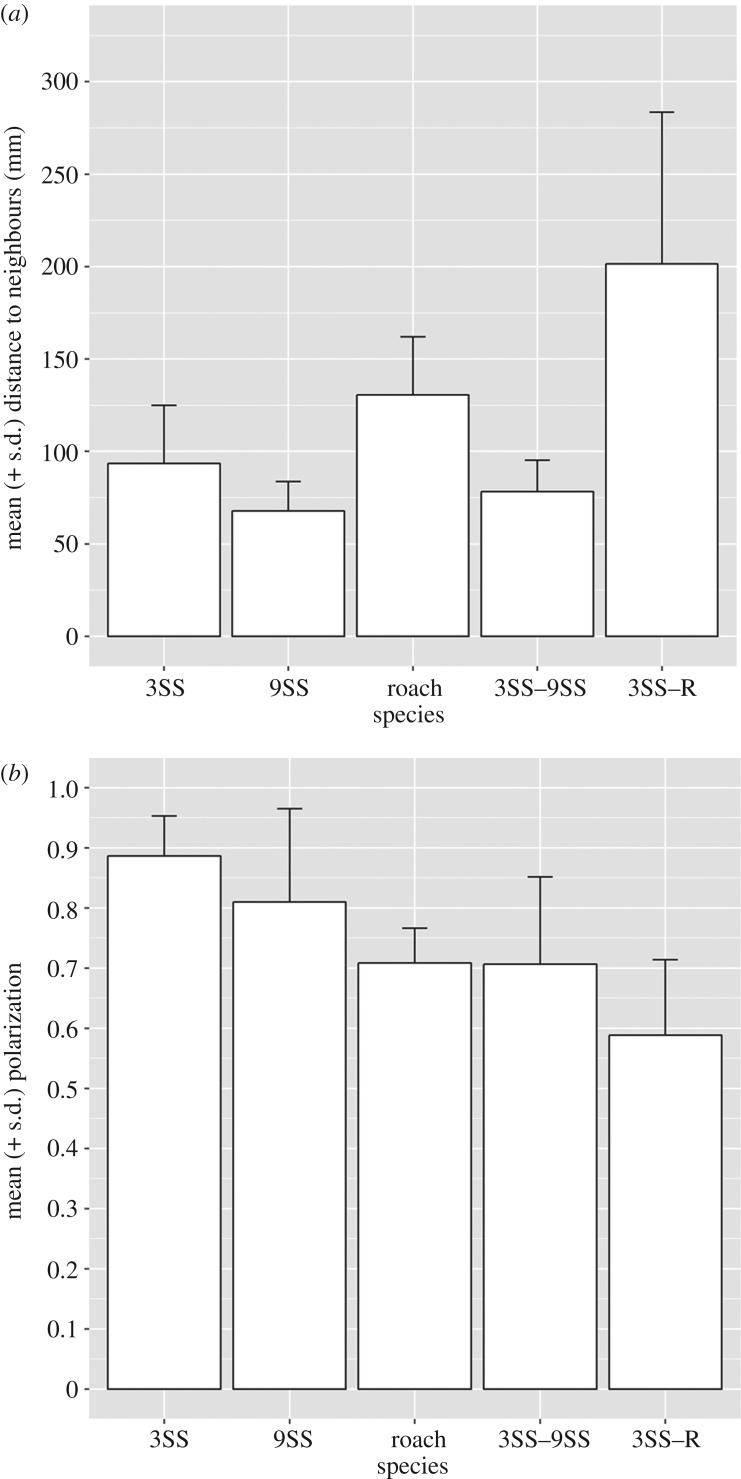
Comparison of (*a*) mean (±s.d.) group cohesion and (*b*) mean (±s.d.) polarization as a function of shoal species composition: 3SS = threespine sticklebacks (*Gasterosteus aculeatus*); 9SS = ninespine sticklebacks (*Pungitius pungitius*); roach = roach (*Rutilus rutilus*); 3SS–9SS = mixed-species shoal comprising threespine and ninespine sticklebacks; 3SS–R = mixed-species shoal comprising threespine sticklebacks and roach.

There was significant variation in group cohesion (linear model: *F*_4,25_ = 10.9, *p* < 0.001; figure 1*a*), polarization (linear model: *F*_4,25_ = 5.64, *p* = 0.002; figure 1*b*), and transfer entropy (linear model: *F*_4,25_ = 3.63, *p* = 0.018) across treatments. Planned contrasts are shown in table 2. Observed mean pairwise transfer entropy for all replicates was significantly greater than the null expectation for all treatments. In each case, each observed value was greater than 1000 surrogate values, indicating a statistically significant directed relationship for all replicates in all treatments.

**Table 2.** Planned non-orthogonal contrasts to examine variance within the dataset between single-species and mixed-species groups, among single-species groups and between mixed-species groups in relation to group cohesion and polarization. 3SS = threespine sticklebacks, 9SS = ninespine sticklebacks, R = roach.

					95% Conf Int		
response variable	contrast	Est	s.e.	*t*	lower	upper	*p*-value
group cohesion	among single-species groups	−0.43	0.02	−3.23	−0.71	−0.15	0.003
	between mixed-species groups	0.38	0.08	4.97	0.22	0.54	<0.001
	3SS, 9SS and mixed 3SS–9SS shoals	−0.01	0.13	−0.04	−0.28	0.27	0.972
	3SS, roach and mixed 3SS–R shoals	0.48	0.13	3.59	0.21	0.76	0.001
polarization	among single-species groups	−0.84	0.3	−2.87	−0.8	−0.58	0.008
	between mixed-species groups	0.18	0.17	1.05	−1.46	−0.24	0.3
	3SS, 9SS and mixed 3SS–9SS shoals	0.76	0.3	2.59	−0.17	0.53	0.016
	3SS, roach and mixed 3SS–R shoals	0.75	0.3	2.54	0.16	1.37	0.018
transfer entropy	among single-species groups	0.01	0.01	1.62	0	0.03	0.12
	between mixed-species groups	−0.01	0.00	−1.55	−0.01	0.02	0.13
	3SS, 9SS and mixed 3SS–9SS shoals	−0.01	0.01	−0.5	−0.02	0.01	0.62
	3SS, roach and mixed 3SS–R shoals	−0.01	0.01	−0.65	−0.02	0.01	0.52

**Figure 2.**
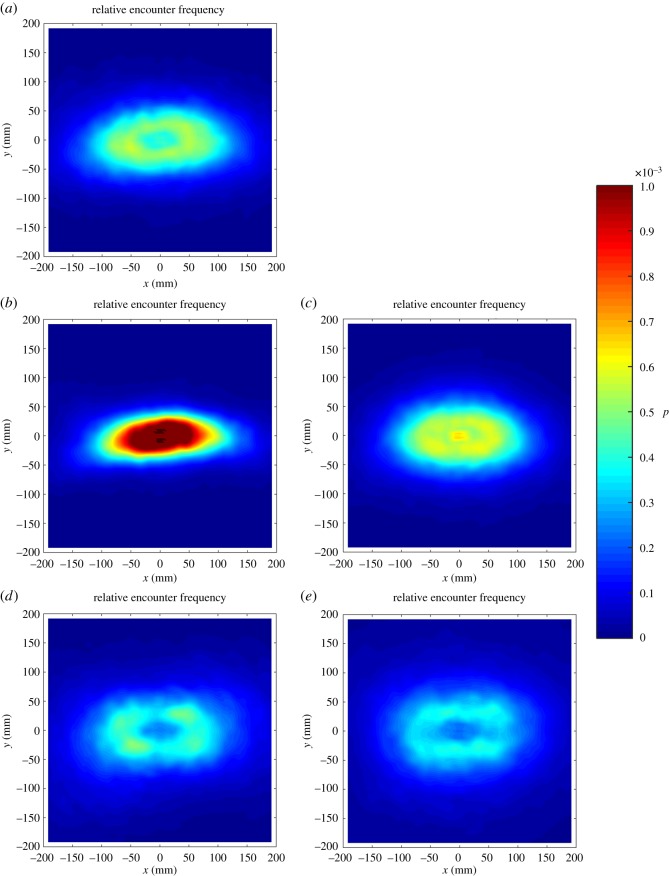
Heat plots showing the relative frequency of near neighbours to a focal individual positioned at the origin and travelling parallel to the positive *x*-axis. Panel (*a*) shows single-species 3SS shoals; (*b*) shows single-species 9SS shoals (*c*) shows mixed 3SS–9SS shoals; (*d*) shows single-species shoals of roach; (*e*) shows mixed 3SS-roach shoals.

**Figure 3.**
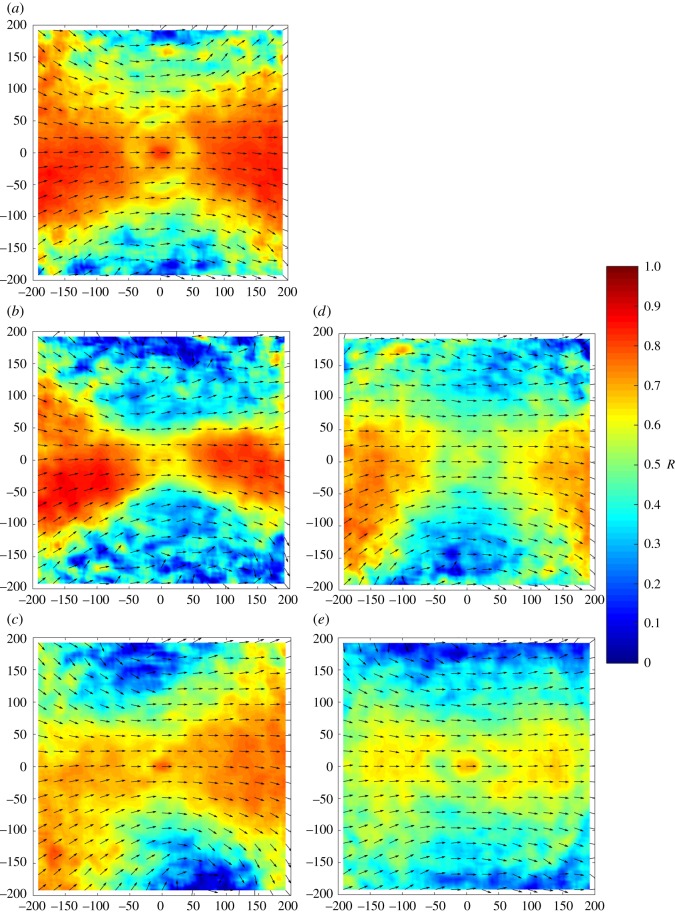
Alignment in direction of motion of near neighbours relative to a focal individual positioned at the origin and travelling parallel to the positive *x*-axis. Arrow shows mean alignment direction of neighbours at this position, while colour shows *R*—a measure of the focus of all relative angles of motion observed about the mean at each relative (*x,y*) coordinate. High values of *R* (closer to 1) indicate greater focus about the mean, whereas lower values indicate lower focus about the mean. Panel (*a*) shows single-species 3SS shoals; (*b*) shows single-species 9SS shoals; (*c*) shows mixed 3SS–9SS shoals; (*d*) shows single-species shoals of roach; (*e*) shows mixed 3SS–R shoals.

Heat plots showing the distribution of near neighbours relative to a focal individual positioned at the origin (*0*,*0*) show a typical elliptical pattern, with a relatively low occurrence in the immediate proximity of the focal individual and peak occurrence of near neighbours at a distance of 1–2 body lengths (figure 2). Heat plots showing the alignment of near neighbours relative to a focal fish at the origin show peak alignment ahead of, as well as behind, that individual, which may be typical of elongated moving groups (figure 3).

Within the mixed-species groups, there was no significant difference between the mean distance of 3SS to conspecifics and their mean distance to 9SS (paired *t*-test: *t*_5_ = −0.372, *p* = 0.725; 95% confidence interval: −14.92–11.15; effect size = 0.164); however, 3SS were located on average significantly closer to conspecifics than to roach (paired *t*-test: *t*_5_ = 3, *p* = 0.03; 95% confidence interval: −331.24–25.75; effect size = 0.8).

There was no significant difference in the mean positions of 3SS versus 9SS sticklebacks (paired *t*-test: *t*_5_ = 2.37, *p* = 0.065; 95% confidence interval: −1.42–0.06; effect size = 0.726), while 3SS were found significantly further towards the front compared to roach in the 3SS–R shoals (paired *t*-test: *t*_5_ = 2.76, *p* = 0.04; 95% confidence interval: 1–0.035; effect size = 0.776).

**Figure 4.**
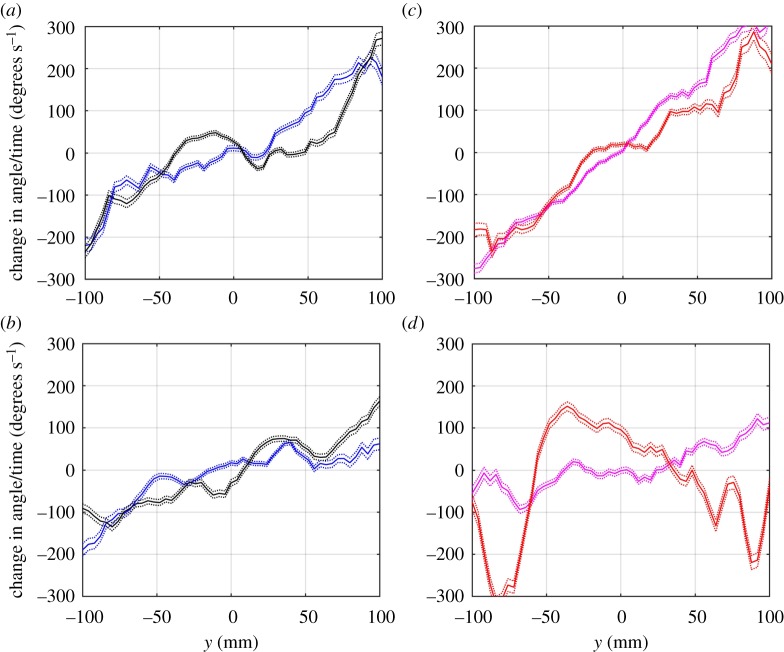
Mean localized turning response (± s.e.) in degrees per second of focal fish to near neighbours positioned to either side of themselves in the mixed-species treatments. The motion of the focal fish is perpendicular to the page, moving towards the reader. Positive changes in angle of motion indicate a turn to the left by the focal individual (relative to its direction of motion), whereas negative changes in angle of motion indicate a turn to the right. Panel (*a*) describes the response of threespine sticklebacks focals to conspecifics (blue curve) and to ninespine sticklebacks (black curve), while panel (*b*) describes the same responses but for the threespine sticklebacks and roach treatment, so that the black curve in this instance represents the response of threespine sticklebacks to roach. Panel (*c*) describes the response of focal ninespine sticklebacks to conspecifics (magenta) and to threespine sticklebacks (red), while panel (*d*) describes the response of focal roach to conspecifics (magenta) and to threespine sticklebacks (red).

The turning response of focal fish in response to near neighbours (figure 4) appears steeper (greater responsiveness in the tendency to turn towards near neighbours) and more coherent (less differentiation in the responses of focal fish to conspecifics versus heterospecifics) in mixed 3SS–9SS shoals than in mixed 3SS–R shoals, which would tend to indicate that the potential for more effective collective motion for groups comprising the two stickleback groups.

**Figure 5.**
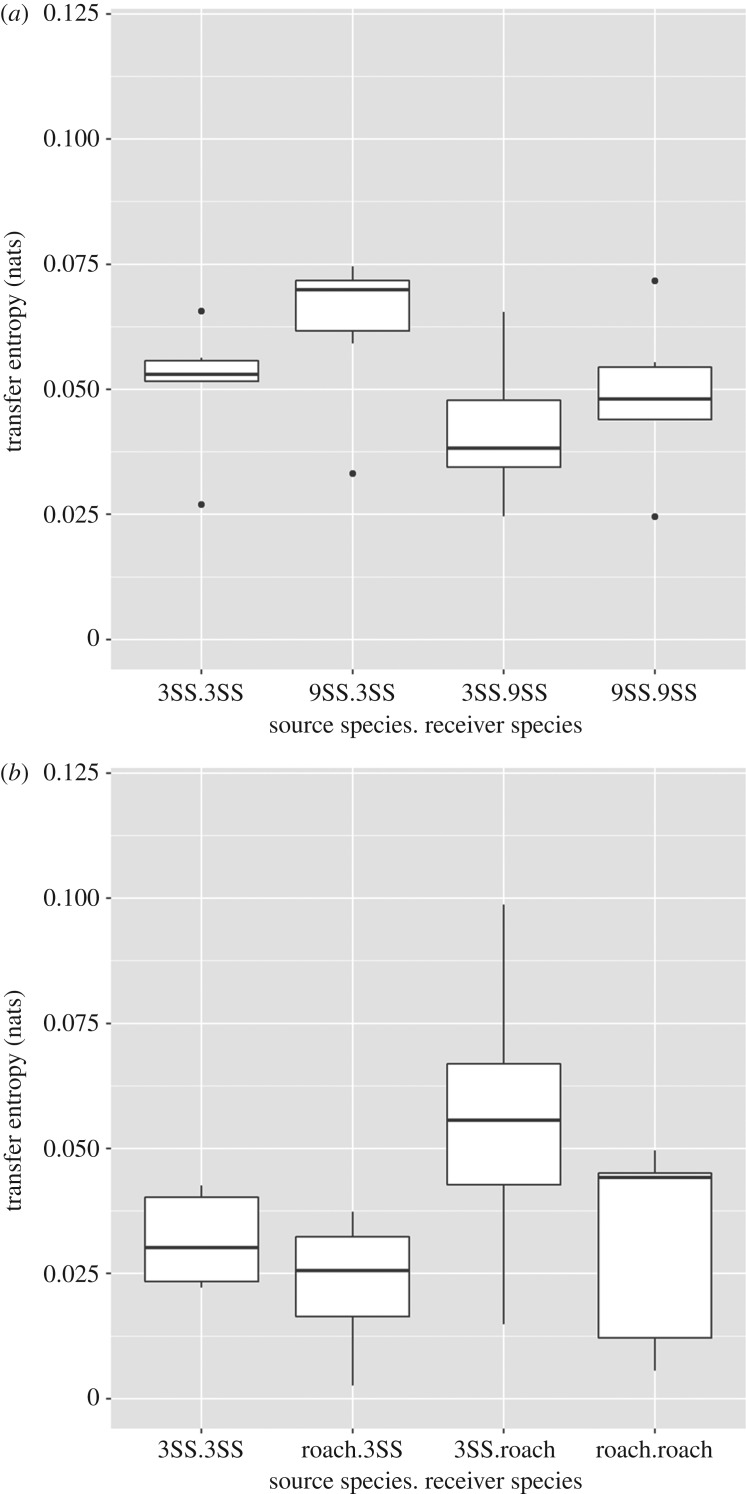
Boxplots to show transfer entropy (nats) in (*a*) the 3SS–9SS mixed-species shoals and (*b*) the 3SS–R mixed-species shoals.

Transfer entropy varied significantly between species in the mixed-species groups (table 3). In the mixed 3SS–9SS groups, information flow was affected by both the identity of the source species as well as according to which species was the receiver (target) species. A similar pattern was evident in the mixed 3SS–R groups with the exception that only the identity of the source species was important, with 3SS more likely to act as information sources (figure 5).

**Table 3.** Differences in mean transfer entropy between source and target species. 3SS = threespine sticklebacks, 9SS = ninespine sticklebacks.

				95% Conf Int		
treatment	information source	*χ*^2^	d.f.	lower	upper	*p*-value
mixed 3SS and 9SS	source species	10.679	1	0.004	0.02	0.001
	target species	16.987	1	−0.017	0.002	<0.001
	source × target interaction	0.831	1	−0.016	0.006	0.362
mixed 3SS and roach	source species	7.223	1	−0.04	0.012	0.007
	target species	2.736	1	0.007	0.044	0.098
	source × target interaction	2.415	1	−0.047	0.002	0.12

## 4. Discussion

Single-species groups of roach and of 3SS were more cohesive and more polarized than mixed-species shoals comprising these two species. By contrast, mixed 3SS–9SS shoals were as cohesive, although not as polarized, as single-species shoals of their constituent species. There was a significant difference across all treatments in information flow, measured as mean transfer entropy. Furthermore, there were significant differences in the mixed-species shoals in relation to the tendency of species to act as the source (to trigger), or as the receiver (to act upon) changes in orientation.

Mixed-species shoals comprising the two stickleback species were no less cohesive than the single-species shoals formed by each of those species, suggesting that the stickleback species can shoal together effectively. However, the mixed 3SS–9SS shoals showed an apparent decrease in polarization relative to the two single-species shoals. In contrast to this, mixed 3SS–R shoals were both less cohesive and less polarized than when each species shoaled individually. Differences in cohesion and in polarization at the group level probably reflect patterns of organization within the group. In mixed 3SS–R shoals, 3SS maintained lower distance to conspecifics and were found in a different part of the shoal, towards the front. These findings suggest that 3SS and roach do not integrate well and thus that mixed-species shoals comprising these two species may be relatively short-lived. Comparative studies on the stability and persistence of mixed-species groups that differ in their constituent member species are lacking and acquiring this kind of basic information would a useful starting point for further research in this area.

Our examinations of patterns of cohesion are broadly supported by the accompanying heat maps, which show high clustering of near neighbours in both stickleback shoals and relatively lower clustering in roach shoals. In comparison to the cohesion seen in the single-species shoals, mixed 3SS–9SS shoals were approximately intermediate to their single-species counterparts, whereas the mixed 3SS–R shoals showed lower clustering than either species on its own. The heat maps describing patterns of polarization in single-species shoals show typically high alignment of the focal fish with group mates immediately in front of and immediately behind the focal fish, consistent with elongated, travelling groups. By contrast, both mixed-species treatments show lower alignment than that observed in the single-species treatments.

Information flow provides a potential mechanistic explanation for these observed patterns. It is important to note, however, that measurements of transfer entropy do not necessarily imply direct causality in terms of information flow between the source (or ‘sender’) and the target (or ‘receiver’) [54]. For example, both parties could be responding independently to some external cue. That said, the measurements of transfer entropy were significantly ‘non-zero’ in all treatments, including the mixed-species groups, and there were no significant decreases in mean pairwise transfer entropy at the group level between single- and mixed-species groups which does suggest information flow both between and within species. Differences in mean pairwise transfer entropy observed between the two species in each of the mixed-species shoals implies that asymmetries in information flow could at least partially account for the reductions in cohesion and polarization seen at the group level. In some cases, the transfer entropy results align with what we see from the other measures, e.g. the higher transfer from 3SS as a source compared to roach correlates with the finding of 3SS being positioned at the front of the shoal. Yet other results of the transfer entropy analysis are not visible in the other measures, e.g. the higher transfers seen from 9SS as a source compared to 3SS in the mixed shoal are not reflected in their relative positions in the shoal. The information transfer analysis thus reveals features of the interactions between species that extend beyond dynamics that were explained otherwise here. Applications of information theoretic measures such as transfer entropy therefore provide a valuable means of quantifying the processes and interactions occurring within animal groups.

What does this mean for mixed-species groups in the wild? Our field observations provide evidence of the occurrence of such groups under natural conditions; however, our controlled experiments in the arena suggest that, in some cases, mixed-species shoals may not be quite so cohesive, or so polarized, as single-species groups. While individuals may obtain some benefit from associating with heterospecifics as well as with conspecifics, mixed-species associations (in this system at least) may be relatively more prone to splitting along species lines, and thus may be more short-lived than their single-species counterparts. It would be valuable therefore to observe shoals in the wild for extended periods to see whether this prediction is correct. More generally, mixed-species groups are common, both with respect to the frequency with which they are observed and their taxonomic distribution, yet the research effort directed towards understanding their functions and underlying social organization is relatively insubstantial compared to that focused upon the dynamics of single-species groups. Under what conditions do mixed-species groups form and how is their frequency in a given environment related to the relative abundance of each of the member species? How generally applicable is the observation from some studies [3,9,15,16,55] that groups tend to be numerically dominated by one or just a few core species with further satellite species occurring in lower numbers? Does this observation have some functional significance, perhaps related to inter- and intraspecific differences in interaction rules, or does it merely reflect the local distribution of member species? Related to this, how do species-specific differences in travel energetics affect group composition in mobile groups? For example Krause *et al.* [16] revealed that size-specific locomotor performance differed between species, and that the composition of mixed-species groups was determined more by matching of swimming speeds across species than by phenotypic matching of shoal members according to body size. Finally, how do external factors such as predation risk or resource distribution affect the underlying mechanics that maintain mixed-species groups? Over the last three decades researchers have made great advances in understanding the functions and organization of single-species animal groups; extending this understanding to encompass mixed-species groups is an obvious next step and, we argue, should be a priority for researchers interested in social behaviour.

## Supplementary Material

Figure S1

## Supplementary Material

Figure S2

## Supplementary Material

Figure S3

## Supplementary Material

Figure S4

## Supplementary Material

Data

## Data accessibility

Data used in the analyses described in this paper are freely available and have been submitted as electronic supplementary information alongside this manuscript.

## References

[1] Ward AJW, Webster MM 2016 Sociality: The behaviour of group-living animals. Berlin, Germany: Springer.

[2] Krause J, Ruxton GD 2002 Living in groups. Oxford, UK: Oxford University Press.

[3] Morse DH 1970 Ecological aspects of some mixed-species foraging flocks of birds. Ecol. Monogr. 40, 119– 168. (10.2307/1942443)

[4] Sullivan KA 1984 Information exploitation by downy woodpeckers in mixed-species flocks. Behaviour 91, 294–311. (10.1163/156853984X00128)

[5] Dolby AS, Grubb TCJ 1998 Benefits to satellite members in mixed-species foraging groups: an experimental analysis. Anim. Behav. 56, 501–509. (10.1006/anbe.1998.0808) 9787042

[6] Satischandra SHK, Kudavidanage EP, Kotagama SW, Goodale E 2007 The benefits of joining mixed-species flocks for a sentinel nuclear species, the greater racket-tailed drongo *Dicrurus paradiseus*. Forktail 23, 145–148.

[7] Goodale E et al. 2009 Regional variation in the composition and structure of mixed-species bird flocks in the Western Ghats and Sri Lanka. Curr. Sci. 97, 647–662.

[8] Sridhar H, Beauchamp G, Shanker K 2009 Why do birds participate in mixed-species foraging flocks? A large-scale synthesis. Anim. Behav. 78, 337–347. (10.1016/j.anbehav.2009.05.008)

[9] Farine DR, Garroway CJ, Sheldon BC 2012 Social network analysis of mixed-species flocks: exploring the structure and evolution of interspecific social behaviour. Anim. Behav. 84, 1271–1277. (10.1016/j.anbehav.2012.08.008)

[10] Jolles JW, King AJ, Manica A, Thornton A 2013 Heterogeneous structure in mixed-species corvid flocks in flight. Anim. Behav. 85, 743–750. (10.1016/j.anbehav.2013.01.015)

[11] Goodale E, Ratnayake CP, Kotagama SW 2014 Vocal mimicry of alarm-associated sounds by a drongo elicits flee and mobbing responses from other species that participate in mixed-species bird flocks. Ethology 120, 266–274. (10.1111/eth.12202)

[12] Stensland E, Angerbjorn A, Berggren P 2003 Mixed species groups in mammals. Mamm. Rev. 33, 205–223. (10.1046/j.1365-2907.2003.00022.x)

[13] Wolf NG 1985 Odd fish abandon mixed-species groups when threatened. Behav. Ecol. Sociobiol. 17, 47–52. (10.1007/BF00299428)

[14] Hoare DJ, Krause J, Peuhkuri N, Godin JGJ 2000 Body size and shoaling in fish. J. Fish Biol. 57, 1351–1366. (10.1111/j.1095-8649.2000.tb02217.x)

[15] Hoare DJ, Ruxton GD, Godin JGJ, Krause J 2000 The social organization of free-ranging fish shoals. Oikos 89, 546–554. (10.1034/j.1600-0706.2000.890314.x)

[16] Krause J, Ward AJW, Jackson AL, Ruxton GD, James R, Currie S 2005 The influence of differential swimming speeds on composition of multi-species fish shoals. J. Fish Biol. 67, 866–872. (10.1111/j.0022-1112.2005.00768.x)

[17] Goodale E, Beauchamp G, Ruxton GD 2017 Mixed-species groups of animals. New York, NY: Academic Press.

[18] Wolters S, Zuberbuhler K 2003 Mixed-species associations of Diana and Campbell's monkeys: the costs and benefits of a forest phenomenon. Behaviour 140, 371–385. (10.1163/156853903321826684)

[19] McGraw WS, Bshary R 2002 Association of terrestrial mangabeys (*Cercocebus atys*) with arboreal monkeys: experimental evidence for the effects of reduced ground predator pressure on habitat use. Int. J. Primatol. 23, 311–325. (10.1023/A:1013883528244)

[20] Webster MM, Ward AJW, Hart PJB 2008 Shoal and prey patch choice by co-occurring fishes and prawns: inter-taxa use of socially transmitted cues. Proc. R. Soc. B 275, 203–208. (10.1098/rspb.2007.1178) PMC2596184 17986436

[21] Farine DR, Aplin LM, Sheldon BC, Hoppitt W 2015 Interspecific social networks promote information transmission in wild songbirds. Proc. R. Soc. B 282, 20172804 (10.1098/rspb.2014.2804) PMC4345451 25673683

[22] Sasvari L 1992 Great tits benefit from feeding in mixed-species flocks: a field experiment. Anim. Behav. 43, 289–296. (10.1016/S0003-3472(05)80224-6)

[23] Hino T 2000 Intraspecific differences in benefits from feeding in mixed-species flocks. J. Avian Biol. 31, 441–446. (10.1034/j.1600-048X.2000.310402.x)

[24] Sridhar H et al. 2012 Positive relationships between association strength and phenotypic similarity characterize the assembly of mixed-species bird flocks worldwide. Am. Nat. 180, 777–790. (10.1086/668012) 23149402

[25] Seppanen JT, Forsman JT, Monkkonen M, Thomson RL 2007 Social information use is a process across time, space, and ecology, reaching heterospecifics. Ecology 88, 1622–1633. (10.1890/06-1757.1) 17645008

[26] Conradt L, Roper TJ 2000 Activity synchrony and social cohesion: a fission-fusion model. Proc. R. Soc. Lond. B 267, 2213–2218. (10.1098/rspb.2000.1271) PMC1690793 11413635

[27] Deutsch A, Theraulaz G, Vicsek T 2012 Collective motion in biological systems. Interface Focus 2, 689–692. (10.1098/rsfs.2012.0048) PMC3499128 24312723

[28] Herbert-Read JE, Perna A, Mann RP, Schaerf TM, Sumpter DJT, Ward AJW 2011 Inferring the rules of interaction of shoaling fish. Proc. Natl Acad. Sci. USA 108, 18 726–18 731. (10.1073/pnas.1109355108) 22065759 PMC3219133

[29] Katz Y, Tunstrom K, Ioannou CC, Huepe C, Couzin ID 2011 Inferring the structure and dynamics of interactions in schooling fish. Proc. Natl Acad. Sci. USA 108, 18 720–18 725. (10.1073/pnas.1107583108) 21795604 PMC3219116

[30] Herbert-Read JE, Buhl J, Hu F, Ward AJW, Sumpter DJT 2015 Initiation and spread of escape waves within animal groups. R. Soc. open sci. 2, 140355 (10.1098/rsos.140355) 26064630 PMC4448869

[31] Schreiber T 2000 Measuring information transfer. Phys. Rev. Lett. 85, 461–464. (10.1103/PhysRevLett.85.461) 10991308

[32] Bossomaier T, Barnett L, Harre M, Lizier J 2016 An introduction to transfer entropy: information flow in complex systems. Berlin, Germany: Springer.

[33] Wang XR, Miller JM, Lizier JT, Prokopenko M, Rossi LF 2012 Quantifying and tracing information cascades in swarms. PLoS ONE 7, e40084 (10.1371/journal.pone.0040084) 22808095 PMC3395630

[34] Orange N, Abaid N 2015 A transfer entropy analysis of leader-follower interactions in flying bats. Eur. Phys. J. Spec. Top. 224, 3279–3293. (10.1140/epjst/e2015-50235-9)

[35] Tomaru T, Murakami H, Niizato T, Nishiyama Y, Sonoda K, Moriyama T, Gunji Y-P 2016 Information transfer in a swarm of soldier crabs. Artif. Life Robot. 21, 177–180. (10.1007/s10015-016-0272-y)

[36] Hu F, Nie LJ, Fu SJ 2015 Information dynamics in the interaction between a prey and a predator fish. Entropy 17, 7230–7241. (10.3390/e17107230)

[37] Crosato E et al. 2017 Informative and misinformative interactions in a school of fish. (http://arxiv.org/abs/1705.01213).

[38] Lord WM, Sun J, Ouellette NT, Bollt EM 2016 Inference of causal information flow in collective animal behavior. IEEE Trans. Mol. Biol. Multi-Scale Commun. 2, 107–116.

[39] Cliff OM, Lizier JT, Wang XR, Wang P, Obst O, Prokopenko M 2017 Quantifying long-range interactions and coherent structure in multi-agent dynamics. Artif. Life 23, 34–57. (10.1162/ARTL_a_00221) 28140630

[40] Butail S, Ladu F, Spinello D, Porfiri M 2014 Information flow in animal-robot interactions. Entropy 16, 1315–1330. (10.3390/e16031315)

[41] Ward AJW, Schaerf TM, Herbert-Read JE, Morrell LJ, Sumpter DJT, Webster MM 2017 Local interactions and global properties of free-ranging stickleback shoals. R. Soc. open sci. 4, 170043 (10.1098/rsos.170043) 28791135 PMC5541530

[42] Pitcher T 1993 Behaviour of teleost fishes, 2nd edn Fish and Fisheries Series 7 London, UK: Chapman & Hall.

[43] Branson K, Robie AA, Bender J, Perona P, Dickinson MH 2009 High-throughput ethomics in large groups of *Drosophila*. Nat. Methods 6, 451–457. (10.1038/nmeth.1328) 19412169 PMC2734963

[44] Schaerf TM, Dillingham PW, Ward AJW 2017 The effects of external cues on individual and collective behavior of shoaling fish. Sci. Adv. 3, e1603201 (10.1126/sciadv.1603201) 28691088 PMC5482554

[45] Cover TM, Thomas JA 2005 Elements of information theory. New York, NY: John Wiley & Sons, Inc.

[46] Kraskov A, Stogbauer H, Grassberger P 2004 Estimating mutual information. Phys. Rev. E 69, 6 (10.1103/PhysRevE.69.066138) 15244698

[47] Lizier JT 2014 JIDT: An information-theoretic toolkit for studying the dynamics of complex systems. Front. Robot. AI 1, 11 (10.3389/frobt.2014.00011)

[48] Garland J, James RG, Bradley E 2016 Leveraging information storage to select forecast-optimal parameters for delay-coordinate reconstructions. Phys. Rev. E 93, 022221 (10.1103/PhysRevE.93.022221) 26986345

[49] Wibral M et al. 2013 Measuring information-transfer delays. PLoS ONE 8, e55809 (10.1371/journal.pone.0055809) 23468850 PMC3585400

[50] Vicente R, Wibral M, Lindner M, Pipa G 2011 Transfer entropy-a model-free measure of effective connectivity for the neurosciences. J. Comput. Neurosci. 30, 45–67. (10.1007/s10827-010-0262-3) 20706781 PMC3040354

[51] Lizier JT, Heinzle J, Horstmann A, Haynes JD, Prokopenko M 2011 Multivariate information-theoretic measures reveal directed information structure and task relevant changes in fMRI connectivity. J. Comput. Neurosci. 30, 85–107. (10.1007/s10827-010-0271-2) 20799057

[52] R Development Core Team. 2011 R: a language and environment for statistical computing. Vienna, Austria: R Foundation for Statistical Computing See https://www.R-project.org/.

[53] Maier R 2015 A (sort of) complete guide to contrasts in R 2015. See https://rstudio-pubs-static.s3.amazonaws.com/65059_586f394d8eb84f84b1baaf56ffb6b47f.html.

[54] Lizier JT, Prokopenko M 2010 Differentiating information transfer and causal effect. Eur. Phys. J. B 73, 605–615. (10.1140/epjb/e2010-00034-5)

[55] Krause J, Hoare DJ, Croft D, Lawrence J, Ward A, Ruxton GD, Godin J-GJ, James R 2000 Fish shoal composition: mechanisms and constraints. Proc. R. Soc. Lond. B 267, 2011–2017. (10.1098/rspb.2000.1243) PMC1690773 11075715

